# Patient-Reported Outcome Measures 2 Years After Standard and Distal Gastric Bypass—a Double-Blind Randomized Controlled Trial

**DOI:** 10.1007/s11695-017-2891-3

**Published:** 2017-09-01

**Authors:** Marius Svanevik, Hilde Risstad, Tor-Ivar Karlsen, Jon A. Kristinsson, Milada Cvancarova Småstuen, Ronette L Kolotkin, Torgeir T Søvik, Rune Sandbu, Tom Mala, Jøran Hjelmesæth

**Affiliations:** 1 Morbid Obesity Center, Vestfold Hospital Trust, Tønsberg, Norway; 2 Institute of Clinical Medicine, University of Oslo, Oslo, Norway; 3 Department of Gastrointestinal Surgery, Vestfold Hospital Trust, Tønsberg, Norway; 4 Department of Endocrinology, Morbid Obesity, and Preventive Medicine, Oslo University Hospital, Oslo, Norway; 5 Oslo and Akershus University College of Applied Science, Oslo, Norway; 6 Quality of Life Consulting, Durham, NC USA; 7 Department of Gastrointestinal Surgery, Oslo University Hospital, Oslo, Norway

**Keywords:** Quality of life, Bariatric surgery, Lifestyle modification, Outcome, Health-related quality of life

## Abstract

**Background:**

The preferred surgical procedure for treating morbid obesity is debated. Patient-reported outcome measures (PROMs) are relevant for evaluation of the optimal bariatric procedure.

**Methods:**

A total of 113 patients with BMI from 50 to 60 were randomly assigned to standard (*n* = 57) or distal (*n* = 56) Roux-en-Y gastric bypass (RYGB). Validated PROMS questionnaires were completed at baseline and 2 years after surgery. Data were analyzed using mixed models for repeated measures and the results are expressed as estimated means and mean changes.

**Results:**

Obesity-related quality of life improved significantly after both procedures, without significant between-group differences (− 0.4 (95% CI = − 8.4, 7.2) points, *p* = 0.88, ES = 0.06). Both groups had significant reductions in the number of weight-related symptoms and symptom distress score, with a mean group difference (95% CI) of 1.4 (− 0.3, 3.3) symptoms and 5.0 (2.9. 12.8) symptom distress score points. There were no between-group differences for uncontrolled eating (22.0 (17.2–26.7) vs. 28.9 (23.3–34.5) points), cognitive restraint (57.4 (52.0–62.7) vs. 62.1 (57.9–66.2) points), and emotional eating (26.8 (20.5–33.1) vs. 32.6 (25.5–39.7) points).

The prevalence of anxiety was 33% after standard and 25% after distal RYGB (*p* = 0.53), and for depression 12 and 9%, respectively (*p* = 0.76).

**Conclusions:**

There were no statistically significant differences between standard and distal RYGB 2 years post surgery regarding weight loss, obesity-related quality of life, weight-related symptoms, anxiety, depression, or eating behavior.

**Trial Registration:**

Clinical Trials.gov number NCT00821197

## Introduction

Bariatric surgery may induce sustained weight loss, improvement of weight-associated comorbidities, and improved health and well-being [1, 2]. However, the preferred surgical procedure is debated, particularly for patients with a BMI of 50 kg/m^2^ or more [3].

Roux-en-Y gastric bypass (RYGB) remains widely applied [4]. Several variants of RYGB exist with remarkably consistent effects on weight loss [5–8]. Changes in size of the pouch [9], stoma [10], and minor changes in the length of the alimentary limb do not seem to impact weight loss [11]. To improve the effectiveness of RYGB, attention has thus been on the length of the common channel or the biliopancreatic limb [12, 13]. The distal RYGB is a variant with a relatively short common channel that may improve weight loss [14].

Most trials assessing bariatric surgery focus on weight loss and resolution of comorbidities. We hypothesized that distal RYGB leads to greater BMI loss 2 years after surgery compared to standard RYGB. However, we found no significant difference in BMI loss between the two procedures [15]. No studies have reported on the effect on health-related quality of life (HRQOL) and well-being, measured by patient-reported outcome measures (PROMs) after distal and proximal RYGB.

PROMs aim to assess the patients’ experience of health and well-being. Such assessment can be performed by using generic- and diagnose-specific questionnaires measuring a broad variety of dimensions of life, e.g., HRQOL, symptom burden, attitudes, and emotions. PROMs are increasingly recognized as important outcome measures after bariatric surgery, but are not systematically applied in trials on bariatric surgery [16].

In the present study, we aimed to compare the effects of standard and distal RYGB on obesity-specific HRQOL, weight-related symptoms, eating behavior, anxiety, and depression.

## Methods

### Trial Design and Participants

The methodology applied in this double-blind, parallel-group randomized controlled trial of standard versus distal RYGB has previously been described [17]. Briefly, all referred patients aged 18 to 60 years with a BMI of 50 to 60 kg/m^2^ were assessed for study enrollment at two Norwegian tertiary care centers between May 2011 and April 2013. Patients with previous bariatric or major abdominal surgery, urolithiasis, chronic liver disease, severe somatic illness, psychiatric disease, or substance abuse were excluded. The 2-year follow-up was completed in May 2015.

Eligible patients were randomly assigned to undergo either standard or distal RYGB using a 1:1 allocation ratio. Patients, follow-up study personnel at the outpatient clinic, and clinicians providing outpatient follow-up were all blinded to treatment allocation.

The study was approved by the Regional Ethics Committees for Medical and Health Research and registered in Clinical Trials: www.clinicaltrials.gov Identifier: NCT00821197. All patients provided written and informed consent.

### Interventions and Follow-up

An antegastric antecolic Roux-en-Y configuration with a gastric pouch of about 25 ml and a biliopancreatic limb of 50 cm were used in both procedures. The standard RYGB had an alimentary limb of 150 cm, whereas the distal RYGB had a common channel of 150 cm. Questionnaires were self-administered and completed at baseline, and follow-up data were collected at scheduled visits 1 and 2 years after surgery.

## Patient-Reported Outcome Measures

### Moorhead Ardelt Quality of Life Questionnaire II (Moorhead-Ardelt)

The Moorhead-Ardelt is a validated obesity-specific instrument measuring postoperative outcomes of self-perceived quality of life [18]. It consists of six domains measured on a 10-point scale from − 0.50 to + 0.50. The domains are added into a sum score ranging from − 3.0 to + 3.0, scoring from a very poor to a very good outcome. A sum score corresponding to good or very well is considered satisfactory. The questionnaire was administered at both baseline, and 1- and 2-year follow-up [18].

### Obesity and Weight-Loss Quality of Life

The validated obesity-specific Obesity and Weight-Loss Quality of Life (OWLQOL) measures feelings and emotions resulting from being obese and trying to lose weight [20]. The instrument consists of 17 statements rated from zero (“not at all”) to six (“a very great deal) on a 7-point scale. The 17 items form a sum scale ranging from 0 to 102, with higher scores indicating better emotional HRQOL. As proposed by scale authors, the scoring syntax converts the scale to 0–100. The questionnaire was administered at both baseline, 1- and 2-year follow-up [19, 20].

### Weight-Related Symptom Measure

The validated obesity-specific Weight-Related Symptom Measure (WRSM) measures 20 symptoms commonly related to being overweight or obese, including foot problems, joint pain, sensitivity to cold, and shortness of breath using two different sets of items [20, 21]. The first set assesses whether or not a patient is experiencing specific symptoms, and the second set rates the level of the distress of the symptoms with values from zero (“not at all”) to six (“bothers a very great deal”). The first set creates an additive scale summing symptoms from 0 to 20, while the second forms a symptom distress scale ranging from 0 to 120. This was administered at both baseline, 1- and 2-year follow-up [19, 20].

### Three-Factor Eating Questionnaire-R 21

The validated generic Three-Factor Eating Questionnaire-R 21 (TFEQ-R21) measures eating behavior and has been validated for use in individuals with obesity [22, 23]. It consists of 21 items comprising three domain scores: (1) uncontrolled eating, assessing the tendency to lose control over eating when feeling hungry or when exposed to external stimuli; (2) cognitive restraint, assessing the conscious restriction of food intake to control body weight or body shape; and (3) emotional eating, assessing overeating related to negative mood states. The domain scores were transformed to 0–100 scales to facilitate comparison; a higher score indicates more uncontrolled, restraint, or emotional eating. This questionnaire was administered only at the 2-year follow-up.

### Hospital Anxiety and Depression Scale

The validated generic Hospital Anxiety and Depression Scale (HADS) measures symptoms of anxiety and depression using 14 items scored from 0 to 3 [24, 25]. It is decomposed into two domains measuring depression (HADS-D) and anxiety (HADS-A), both consisting of seven items yielding a score from 0 to 21. Norwegian normative data are available [26]. A cutoff point of > 8 yields an adequate sensitivity and specificity for clinically relevant symptoms of depression or anxiety [27]. The form was administered only at the 2-year follow-up.

#### Statistical Analyses

Continuous variables are described with mean and standard deviation (SD), categorical variables with counts and percentages. Possible differences between groups regarding all continuous outcomes with repeated measurements were modeled using linear mixed models for repeated measurements with unstructured covariance matrix. Each model contained fixed effects for treatment, time (measured in weeks after surgery), treatment × time interaction, and a random intercept. Based on these linear mixed models, we estimated mean treatment group values with 95% confidence intervals (CIs) for all time points: baseline, 1, and 2 years after surgery. In addition, we estimated the mean within-group changes from baseline to 2 years and the between-group difference in change from baseline to 2 years.

Possible differences between treatment groups regarding PROMs available at 2 years only (HADS and TFEQ) were assessed with independent samples *t* test and Fisher’s exact test. Crude associations between pairs of categorical variables were assessed using Fischer’s exact test when appropriate. Standardized effect sizes (ES) were estimated from independent samples *t* test as mean change from baseline divided by the pooled standard deviation of the baseline score; we used estimated values from the mixed model when possible. An ES below 0.2 was considered trivial, 0.2 to 0.49 small, 0.50 to 0.79 moderate, and 0.80 and greater large [28]. All tests were two-sided and *p* values < 0.05 were considered statistically significant.

IBM SPSS Statistics for Windows, Version 21.0, was used for the statistical analyses.

#### Missing Data

Missing values were not imputed, except where the scoring guidelines for OWLQOL and WRSM described how missing data should be handled. Mixed model estimations do not require full data sets and use all available data to estimate the results, and the covariance matrix handles missing data without requirements for imputation.

## Results

A total of 113 patients received the allocated treatment: 57 standard and 56 distal RYGBs. Baseline demographics, education, employment, and anthropometrics were comparable between groups (Table 1). The follow-up rate was 97% (*n* = 110). Table 2 shows completion rates of PROMs at all study visits with an overall completion rate of 95% on all forms.

**Table 1:** Observed patient characteristics at baseline by treatment group

	Standard (*n* = 57)	Distal (*n* = 56)
Demographics		
Age, years^a^	38.2 (9.2)	41.3 (8.3)
Gender, female^b^	36 (63)	37 (66)
Ethnicity, caucasian^b^	57 (100)	55 (98)
Education		
< 9 years^b^	15 (26)	15 (27)
9–12 years^b^	29 (51)	29 (52)
> 12 years^b^	13 (23)	12 (21)
Working status		
Working/student^b^	38 (67)	32 (57)
Sickleave^b^	1 (2)	1 (2)
Unemployed^b^	5 (9)	3 (5)
Disability^b^	2 (4)	3 (5)
Temporary disability^b^	11 (19)	17 (30)
Anthropometrics		
Height, cm^a^	173 (10)	171 (10)
Weight, kg^a^	160.2 (19.9)	157.4 (17.3)
BMI, kg/m^2a^	53.3 (2.6)	53.6 (3.3)
Waist circumference, cm^a^	146 (14)	144 (11)

^a^Data are shown as mean (SD)

^b^Data are shown as no. (%)

**Table 2:** Completion of patient-reported outcome measure questionnaires at all time points

	Baseline (113 patients)		1 year (111 patients)		2 years (110 patients)	
Obesity and Weight-Loss Quality of Life	Complete	112 (99%)	Complete	110 (99%)	Complete	110 (100%)
	Incomplete	0 (0%)	Incomplete	0 (0%)	Incomplete	0 (0%)
	Missing	1 (1%)	Missing	1 (1%)	Missing	0 (0%)
Weight Related Symptom Measure	Complete	91 (81%)	Complete	98 (88%)	Complete	105 (95%)
	Incomplete	22 (19%)	Incomplete	13 (12%)	Incomplete	5 (5%)
	Missing	0 (0%)	Missing	0 (0%)	Missing	0 (0%)
Moorhead Ardelt Quality of Life Questionnaire II	Complete	110 (97%)	Complete	108 (97%)	Complete	107 (97%)
	Incomplete	1 (1%)	Incomplete	1 (1%)	Incomplete	2 (2%)
	Missing	2 (2%)	Missing	2 (2%)	Missing	1 (1%)
Hospital and Anxiety Depression Scale	n/a		n/a		Complete	109 (99%)
					Incomplete	0 (0%)
					Missing	1 (1%)
Three-Factor Eating Questionnaire-R21	n/a		n/a		Complete	108 (98%)
					Incomplete	1 (1%)
					Missing	1 (1%)

Number of patients and percentage of patients attending follow-up

*n/a* not available

All scales had satisfactory internal consistency with Cronbach’s alpha coefficients > 0.7.

As previously reported at 2 years, the total BMI loss was 17.8 (95% CI, 16.9–18.6) kg/m^2^ after standard and 17.2 (95% CI, 16.3–18.0) kg/m^2^ after distal, with no significant between-group differences (*p* = 0.32). For general health-related quality of life, the physical summary score was improved and the mental summary score was unchanged for both surgical groups, with no significant between-group difference [15].

Table 3 shows baseline, 1-, and 2-year results as well as estimated within-group changes and between-group differences in PROMS concerning obesity-specific HRQOL and weight-related symptoms.

**Table 3:** Estimated mean scores in WRSM, OWLQOL, and Moorhead Ardelt at baseline and 2 years after standard and distal gastric bypass

	Mean (95% CI)		Within-group change		Between-group difference in change	
	Baseline	2 years	Mean (95% CI)	*p* value	Mean (95% CI)	*p* value
WRSM						
Symptom count						
Standard	9.4 (8.2, 10.6)	7.0 (5.7, 8.3)	− 2.4 (− 1.2, − 3.7)	< 0.001		
Distal	10.2 (8.9, 11.4)	6.4 (5.1, 7.6)	− 3.8 (− 2.4, − 5.2)	< 0.001	1.4 (− 0.3, 3.3)	0.14
Symptom distress						
Standard	32.6 (26.8, 38.4)	20.0 (15.6, 24.4)	− 12.7 (− 7.2, − 18.2)	< 0.001		
Distal	35.8 (29.9, 41.7)	18.2 (13.8, 22.7)	− 17.6 (− 11.8, − 23.4)	< 0.001	5.0 (− 2.9, 12.8)	0.21
OWLQOL						
Standard	37.7 (32.4, 42.9)	77.4 (72.6, 82.2)	39.8 (34.9, 44.7)	< 0.001		
Distal	35.2 (29.9, 40.5)	74.3 (69.5, 79.2)	39.1 (32.9, 45.3)	< 0.001	− 0.4 (− 8.4, 7.2)	0.88
Moorhead Ardelt						
General self-esteem						
Standard	0.17 (0.12, 0.22)	0.26 (0.20, 0.32)	0.09 (0.03, 0.15)	< 0.01		
Distal	0.17 (0.12, 0.22)	0.21 (0.15, 0.26)	0.04 (− 0.03, 0.10)	0.25	− 0.06 (− 0.14, 0.03)	0.18
Physical activity						
Standard	0.04 (− 0.02, 0.11)	0.21 (0.16, 0.27)	0.17 (0.11, 0.23)	< 0.001		
Distal	0.01 (− 0.06, 0.08)	0.19 (0.13, 0.25)	0.17 (0.10, 0.25)	< 0.001	0.01 (− 0.09, 0,10)	0.91
Social contacts						
Standard	0.12 (0.05, 0.19)	0.20 (0.14, 0.26)	0.08 (0.02, 0.15)	0.02		
Distal	0.15 (0.08, 0.22)	0.22 (0.16, 0.28)	0.07 (− 0.01, 0.15)	0.07	− 0.01 (− 0.11, 0.10)	0.90
Work satisfaction						
Standard	0.12 (0.02, 0.21)	0.19 (0.09, 0.29)	0.07 (− 0.02, 0.16)	0.14		
Distal	0.04 (− 0.06, 0.13)	0.16 (0.07, 0.26)	0.12 (0.04, 0.20)	< 0.01	0.05 (− 0.07, 0.18)	0.40
Sexual pleasure						
Standard	− 0.08 (− 0.17, 0.00)	0.07 (− 0.02, 0.16)	0.15 (0.06, 0.24)	< 0.01		
Distal	− 0.17 (− 0.26, − 0,08)	0,06 (− 0.02, 0.15)	0.23 (0.13, 0.33)	< 0.001	0.08 (− 0.04, 0.21)	0.20
Eating behavior						
Standard	0.11 (0.05, 0.16)	0.20 (0.14, 0.26)	0.09 (0.02, 0.17)	0.02		
Distal	0.04 (− 0.02, 0.09)	0.15 (0.09, 0.21)	0.11 (0.03, 0.19)	< 0.01	0.02 (− 0.09, 0.13)	0.75
Total score						
Standard	0.47 (0.24, 0.71)	1.14 (0.85, 1.43)	0.67 (0.38, 0.95)	< 0.001		
Distal	0.24 (0.00, 0.48)	0.99 (0.71, 1.28)	0.75 (0.49, 1.01)	< 0.001	0.09 (− 0.29, 0.47)	0.64

*WRSM* Weight-Related Symptom Measure, *OWLQOL* Obesity and Weight-Loss Quality of Life, *Moorhead-Ardelt* Moorhead-Ardelt Quality of Life Questionnaire II

### Obesity-Specific HRQOL

Obesity-related quality of life (OWLQOL) improved significantly in both groups, with no significant between-group differences.

Self-perceived quality of life improved significantly for all dimensions except for the work-related dimension after standard RYGB, and general self-esteem after distal RYGB. At baseline, 23% of patients reported a good to very good quality of life while 2 years after surgery, this increased to 51% of patients, with no difference between groups (Table 4).

**Table 4:** Comparison of Moorhead Ardelt II QOL outcomes at baseline and 2 years

Groups	Baseline quality of life						2-year quality of life					
	Very poor	Poor	Fair	Good^a^	Very good^a^	Total satisfactory	Very poor	Poor	Fair	Good^a^	Very good^a^	Total satisfactory
Standard RYGB	0	0	43	10	2	12	0	2	24	13	12	26
Distal RYGB	0	5	36	12	1	13	0	2	25	22	6	28

Number of patients

^a^Satisfactory outcomes

### Weight-Related Symptoms

Patients in both groups experienced a significant reduction in the number of weight-related symptoms and symptom distress score with no significant difference between the groups.

**Fig. 1:**
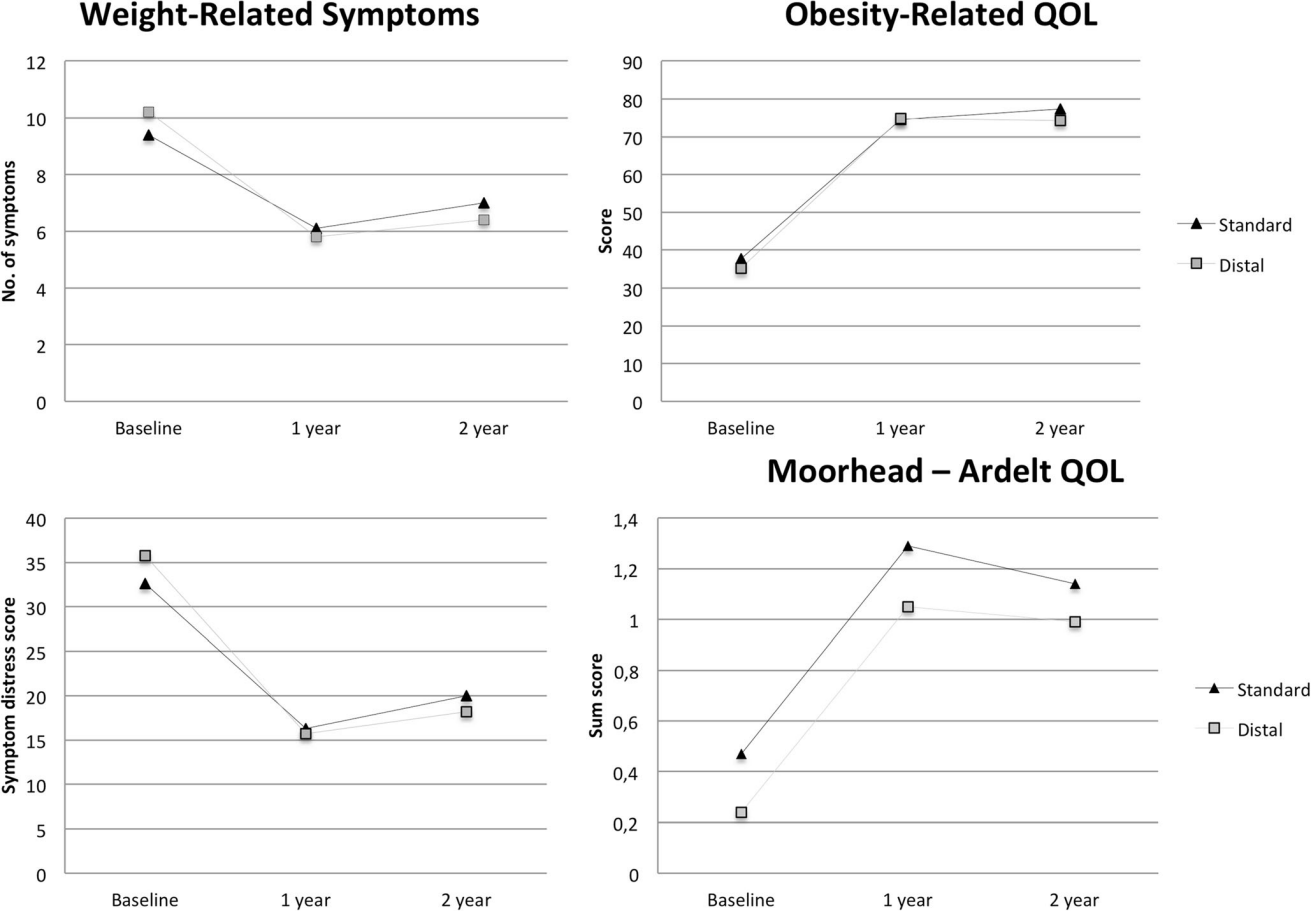
Modeled changes in obesity-specific quality of life and weight-related symptoms after standard and distal RYGB

Most of the improvement in HRQOL and weight-related symptoms occurred during the first year, with only small changes between 1 and 2 years after surgery. Figure 1 shows estimated changes in obesity-specific HRQOL and weight-related symptoms over time.

### Eating Behavior

The mean (95% CI) eating behavior scores did not differ significantly between groups after surgery: uncontrolled eating after standard RYGB (22.0 (17.2–26.7)) vs. distal RYGB (28.9 (23.3–34.5), *p* = 0.06), cognitive restraint (57.4 (52.0–62.7) vs. 62.1 (57.9–66.2) points, *p* = 0.16), and emotional eating (26.8 (20.5–33.1) vs. 32.6 (25.5–39.7) points, *p* = 0.22).

### Anxiety and Depression

The mean (SD) scores at 2 years for anxiety (HADS-A) were 5.2 (4.1–6.4) points for standard and 5.1 (4.0–6.1) points for distal RYGB, respectively (*p* = 0.81), and the prevalence of clinically relevant anxiety was 22% after standard and 11% after distal RYGB (*p* = 0.13). The mean (SD) depression scores were 2.8 (1.8–3.8) points for standard and 2.1 (1.3–3.0) points for distal (*p* = 0.32), and the prevalence of clinically relevant depression was 9% after standard and 5% after distal (*p* = 0.49).

## Discussion

We found improvements in most PROMs after both standard and distal RYGB, but no significant differences between groups after surgery in regard to obesity-specific HRQOL, weight-related symptoms, anxiety and depression, or eating behavior. There was comparable weight loss between the two groups, and we suspect the amount of weight lost could be a major determinant of improvement in HRQOL and other PROMs after bariatric surgery [13].

To the best of our knowledge, this is the first study assessing changes in HRQOL, health, and well-being after distal RYGB. Studies comparing RYGB with more radical surgical procedures such as duodenal switch have shown comparable results on change in HRQOL, despite increased weight loss and more malabsorption after duodenal switch [29].

The improvement in obesity-specific quality of life is comparable to other studies on RYGB [21]. When looking at effect sizes, there was a small difference (ES = 0.26) for improving weight-related symptoms in favor of distal RYGB; however, the implication of this finding is uncertain.

To the best of our knowledge, this is the first study to assess anxiety and depression when comparing standard and distal RYGB, but the scores for anxiety and depression may be compared to the normal population [26] and studies of other bariatric procedures. The mean scores of anxiety are higher than the population norm of obese patients (BMI > 35 kg/m^2^). Aasprang et al. published a study on duodenal switch showing improved anxiety from baseline to 2 years and a worsening of symptoms from 2 to 5 years. The mean (95% CI) HADS-A at 2 years in their study was 4.7 (3.7–5.8), slightly lower but comparable to our 2-year results and the scores for anxiety are comparable to their 2-year results [30]. Both results are higher than the Norwegian population for BMI > 35 kg/m^2^, but high-quality data from patients in the BMI 50–60 kg/m^2^ range is lacking. Other studies have shown improvement in depression after bariatric surgery [31], and both our groups have mean scores comparable to a non-obese population. Long-term follow-up is needed to adequately study this.

Previous studies describe significant improvements in uncontrolled and emotional eating after both RYGB and duodenal switch, but with no significant differences between groups [32], which is in line with our observations. Previous studies have not shown significant changes in cognitive restraint, and our results are slightly higher for both proximal and distal RYGB than those published by Søvik et al. Although non-significant (*p* = 0.06), the mean uncontrolled eating score was slightly higher in the distal RYGB group than in the standard RYGB group in the present study. The lack of baseline data makes the clinical implication of this finding uncertain.

Strengths of this study include the double-blind randomized controlled design and standardized surgical procedures. The patients had a BMI in the 50–60 kg/m^2^ range, creating a homogenous population. Nearly all patients attended follow-up, and the completion rates of questionnaires were high. All patients were recruited from two public hospitals, and treatment provided was independent of health insurance and personal finance.

An important limitation is that evaluation of PROMS was a secondary endpoint. This might induce sample size issues such as type II errors. To evaluate potential differences, we calculated effect sizes to estimate any potentially undetected differences. Only some of the effect sizes are small; all others are trivial suggesting negligible differences. Another limitation is that eating behavior, anxiety, and depression were only measured at 2 years, making comparison of changes impossible. Despite the randomized controlled design, small random differences cannot be excluded.

Standardized reporting of PROMs is gaining more attention and may help patients and clinicians choose appropriate procedures. However, several of the obesity-specific measures of HRQOL are in fact weight dependent and might not differentiate adequately between the adverse effects of surgical procedures. Relevant differences between the procedures with regard to gastrointestinal symptoms and eating quality as well as behavior and specific adverse events are not adequately covered by existing PROMs. The GQLI index measures gastrointestinal symptoms with regard to quality of life [33], but does not explore other post-bariatric problems such as dumping and postprandial hypoglycemia. The development of a symptom measure focusing exclusively on these problems might help clinicians and patients differentiate between different bariatric procedures. However, if differences are subtle and not detectable by existing PROMS, the impact is likely to be small.

The greatest improvement in quality of life and resolution of weight-specific symptoms occur during the first year after bariatric surgery during the greatest weight loss. Weight loss may thus be an important determinant for improvement of quality of life. However, assuming that greater weight loss would improve quality of life even more is not so certain. It could be that the improvement is mostly due to the initial weight loss, and that further weight loss would not lead to further improvement in quality of life. In a study of duodenal switch and RYGB, the increased weight loss after duodenal switch did not translate into increased quality of life [34]. The large initial weight loss may decrease the patients’ feeling of stigma and increase a feeling of normality and this trumps some of the adverse effects of bariatric surgery, at least in short term. Studies have shown that 5-year HRQOL decreased significantly when compared with the scores 1–2 years post surgically [16]. Thus, the 2-year span may not be a sufficient length to detect clinically relevant differences in PROMs.

Our findings cannot be extrapolated to other variants of distal RYGB that might lead to more malabsorption and greater weight loss. At 2 years, we find no evidence to support the use of distal RYGB; however, evaluations following prolonged follow-up may reveal other findings.

## Conclusion

In patients with BMI 50–60 kg/m^2^, both standard and distal RYGB lead to sustained weight loss and improved HRQOL 2 years after surgery. We found no significant differences between the two procedures in regard to obesity-specific HRQOL, weight-related symptoms, anxiety and depression, and eating behavior. Standard RYGB continues to be our first choice in treating patients with BMI above 50 kg/m^2^.
